# CoViD-19 effects on social-emotional development: impact of early intervention

**DOI:** 10.6026/97320630019889

**Published:** 2023-09-30

**Authors:** Michelle Rodriguez-Monge, Iglesias-Peña Isabela, Francesco Chiappelli

**Affiliations:** 1 Costa Rican Institute of Clinical Research, San José, Costa Rica; Stars Therapy Services, San Diego, CA 91913; 2 Boston Children's Martha Eliot - Early Intervention Program, Boston Children's Hospital, Boston, MA 02130,USA; 3 Dental Group of Sherman Oaks, CA 91403, USA(www.oliviacajulisdds.com); 4 Center for the Health Sciences, UCLA, Los Angeles, CA; 90095

**Keywords:** CoViD-19, Social and emotional development, Early Intervention (EI), Artificial Intelligence (AI), Critical Age hypothesis

## Abstract

Age-appropriate development of social and emotional skills is challenging to a child under standard conditions. The CoVID-19 pandemic has likely influenced the development of social, emotional, and communicative skills. Factors like prolonged lockdowns, restricted peer interactions, and mandatory mask-wearing may have hindered children's ability to learn facial expressions and nonverbal cues. The research evidence discussed in this paper confirms that proposition, and examines in further depth the potential impact of the CoViD-19 pandemic. We also discuss groundwork evidence-based early intervention (EI) practices designed to mitigate the negative effects these unprecedented circumstances may have led to, and how tele-medicine alternatives and Artificial intelligence (AI) can expedite interventional childhood plans. The role of bioinformatics is vital in the compilation and analysis of the vast research in this piece related to CoViD-19, serving as a profound search tool for future research endeavors focused on understanding the long term effects of the pandemic.

## Background:

Child development is an intricate and multifaceted process, which can be assessed through various approaches and domains. The use of developmental milestones is a widely recognized approach. Developmental milestones are age-specific abilities or behaviors children will typically achieve as they develop. Age-specific milestones can be organized into several categories and subcategories along certain distinct developmental domains, such as gross motor activity, and fine motor mobility. The latter, includes self-care, communication, namely speech and language mastery, as well as nonverbal communication, and lastly cognitive and socio-emotional skills [1]. The rate of acquisition of appropriate social and emotional abilities is essential for child growth and development as it lays the foundation of the maturing individual's overall wellbeing and success in multiple complex environments. According to Yates and collaborators (2008), social and emotional skills are best defined as the developing capacity of the child from birth through 5 years of age to form close and secure adult and peer relationships; experience, regulate, and express emotions in socially and culturally appropriate ways, and to explore the environment and learn all in the context of family, community and culture [2]. The classification of a developmental delay may include categorizing it according to the time of appearance (e.g., prenatal, postnatal, perinatal, and other), or severity (i.e., mild, moderate, severe, and significant) [3]. To be clear, mild, moderate, and severe developmental delays are evaluated in the primary care setting, with mild developmental delay being evident when a child's functional age is 33% below its chronological age. Moderate developmental delay is diagnosed when a child's functional age is 34% of chronological age. Severe developmental delays are noted when a child's functional age is 66% below chronological age, and significant developmental delays are recognized when the performance of a child is at two or more standard deviations below the mean of age-appropriate standardized more-referenced testing [3]. The etiology of developmental delay is usually multifactorial, along genetic, environmental and or psychosocial factors, and most of the cases are idiopathic. Some examples are Fragile X syndrome, Down syndrome, early maternal infections, maternal stress, prematurity, hypoxic-systemic encephalopathy, maltreatment, poverty and malnutrition [4]. A recent systematic review established that societal adversity affects the child's brain development, multiple body systems, and the physiologic, consequently portraying signs of socio-emotional manifestations in childhood [5]. Taken together, these lines of evidence lead to the proposition that the mandatory lockdown during the CoViD-19 pandemic precluded children from essential social interactions, and the mandatory mask wearing similarly also prevented children from acquiring decoding skills of facial recognition and nonverbal communication. This may have been among the most significant societal correlates that may have impaired age-dependent normal acquisition of social and emotional skills during the CoViD-19 pandemic. Early intervention (EI) programs strive to increase a child's potential from birth to 3 years of life by providing families with additional support and guidance in their child's overall development, and serves as a school-readiness tool to prepare children for the academic setting. Public Law 99-457 (1986 revision) recommended EI to be accessible to all families, and enforced in every States free- or low-cost to ensure support for families with infants experiencing developmental impairments from early infancy, rather than wait until the child's third birthday to obtain services, as was customary until 1986. Public Law 99-457 mandated EI services to provide regular evaluation of the child's development. Nonetheless, criteria to become eligible for EI services vary by state and no doctor referral is required. It is possible and even probable that targeted EI programs could counter CoViD-19- related disadvantages in the children's acquisition of appropriate social and emotional abilities. These EI programs must be developed, tested, applied and evaluated promptly, because the developmental delays brought about by the pandemic are timely and critical. We propose that new and improved EI programs must be developed in the spirit of PL99-457, should entail timely diagnosis, and be targeted provision of catered assistance to the identified children with diagnosed developmental delays [6].

## Methodology:

EI is a highly effective interventional approach for supporting and recovering children who experience developmental delays. Before the CoViD-19 pandemic, 43% of children less than 5 years old were at risk of not achieving their developmental potential. Evidence shows this percentage has increased [7] in the aftermath of the pandemic, and that individualized approaches to aiding a child's specific challenges can help recover the social-emotional and communicative delays in the afflicted children [8. EI comes into play when addressing these challenges because young children tend to exhibit greater neuroplasticity, underlying their ability to learn. Optimizing EI into everyday evaluations may overcome the waitlist crisis for autism evaluations. In fact, promising data suggest that Artificial Intelligence (AI) services in the field of EI can improve the ability to detect or rule out an autism diagnosis, accelerating the provision of interventional services for those with developmental challenges such as social-emotional [9]. AI is also expected to prevent any misdiagnosis related to insufficient training in personnel and lack of readiness, aiming towards a more individualized intervention plan for each child [10]. Increasing the additional support provided to children who are vulnerable to developmental challenges will help decrease the effects of CoViD-19. Transitioning to remote visitations and tele-medicine support may lead to fewer adverse effects [11]. Rather than depriving the child of EI time, utilizing synchronous virtual delivery formats for interventions may yield an increase in child compliance rate, a decrease in behavior problems, and a reduction in parental stress [12]. In brief, it is possible and even probable that novel and improved EI practices by implementing AI and providing additional support to families will help mitigate the effects of this unprecedented time.

## Discussion:

The CoViD-19 pandemic has had far-reaching consequences on young children, many of whom experienced social deprivations. Mask-wearing impaired children's developmental growth and negatively affected language development, literacy acquisition, learning and cognitive development, and emotion recognition processes. There is significant interconnectedness between language development and the child's social emotional growth, suggesting that the inability to observe verbal cues and facial expressions affects emotion recognition processes. The use of masks in children and teachers has led to the child's inability to develop social-emotional skills, causing an inevitable isolation between teachers and students, and a decrease in peer interaction [13]. Evidence supports that the use of masks has affected communication fluency in young children during the pandemic and beyond, raising concerns that align with the Critical Age hypothesis, suggesting that the crucial age range, in which language learning is met, is essential for both language and social-emotional growth [14]. Convergent evidence shows that children susceptible to developmental delays experienced profound negative effects on their social-emotional growth. Vulnerability and susceptibility to drastic change influenced the truncation of social-emotional growth and communicative development in young children since the brain architecture continues to rapidly develop and is highly sensitive to environmental adversity [7]. Systematic reviews investigated normally developing children in comparison to those with special education needs or developmental delays. Data showed that the risk of child psychosocial problems was higher in children with special education needs, developmental delays, and/or those diagnosed with acute or chronic diseases [15], including autism spectrum disorder. The following points further explain why children with developmental delays, special needs, and chronic diseases are more susceptible to adverse impacts throughout their development.

[1] Special education children, and children suspected of suffering from neurodevelopmental disorders are more susceptible to experiencing difficulties with social communication [16]. Children with special needs, including autistic children, experience anxiety when their routine is altered and when facing unfamiliar situations or environments [17]. Research evidence indicates that 70% of participants were diagnosed with at least one comorbid disorder. In fact, the most common diagnoses were social anxiety disorder, ADHD, and oppositional defiant disorder. This population usually experiences delays in their social-emotional and developmental growth [18]. The data suggest that children with special needs and developmental delays are particularly vulnerable to social emotional and developmental challenges, enforced by the use of masks and social isolation.

[2] When the CoViD-19 lockdown started, young children who were receiving EI were forced to pause their therapy schedule and follow strict isolation guidelines, leading to significant loss of access to crucial resources, and effects on the progress of the child's developmental journey. This placed these children in danger of being severely impacted in their social communicative growth and development. It is not surprising that the effects of CoViD-19 on children with developmental delays or/and special services, include a significant regression in social and communication skills, attributable largely to the lack of intervention and social interactions [19].

[3] Parents with children diagnosed with chronic conditions are under greater psycho-emotional stress as their childcare is more demanding than children with no developmental delay. Isolation policies enforced during the CoViD-19 pandemic altered the way families sought and received support from their extended family members. The closure of health care facilities caused an imbalance in the family dynamic in charge of supporting the child in need [8]. Taken together, the absence of emotional support for parents resulted in an increase in their stress levels, which in turn negatively impacted the social-emotional development of the child [8,20]. Mental health effects, and social setbacks and detriments observed in children with chronic conditions due to the CoViD-19 lockdown will be further compiled and analyzed for future months or years following the pandemic (Rodriguez-Monge et al., in preparation).

## Conclusion:

The impact of public health restrictions during the response to the CoViD-19 pandemic on early social-emotional development was discussed in this writing. Taken together, the evidence at hand underscores the critical significance of EI programs in mitigating the adverse effects on both vulnerable children, and on children who were not initially categorized as vulnerable but have nonetheless experienced similar detrimental outcomes. With limited social interactions, disrupted routines, and imposed lockdowns during the pandemic, children encountered unusual early social, cognitive and emotional development challenges. These factors combined with the stress experienced by parents and the caregivers dismantled a never-ending list of obstacles for young children's ability to develop social-emotional skills. The data to date taken together confirms the scope and extent of children's social-emotional development delay, the lack of interactions and stressors during the CoViD-19 pandemic, and show that optimized EI services could and did potentially fill in the gap, allowing these skills to develop accordingly in children receiving the service. Research findings also suggest that EI programs combined with AI can accelerate the turnover of delivering critical intervention for children in need in a timely manner. These enhanced early care services can yield great improvements for disadvantaged children during the pandemic, and ought not to be discontinued.
